# Modelling survival data to account for model uncertainty: a single model or model averaging?

**DOI:** 10.1186/2193-1801-2-665

**Published:** 2013-12-11

**Authors:** Sri Astuti Thamrin, James M McGree, Kerrie L Mengersen

**Affiliations:** Mathematical Sciences, Queensland University of Technology, GPO Box 2434, 4001 Brisbane, Queensland Australia; Mathematics Department, Hasanuddin University, Jl. Perintis Kemerdekaan Km 10, 90245 Makassar, South Sulawesi Indonesia

**Keywords:** Bayesian modelling, Bayesian model averaging, Cure model, Markov Chain Monte Carlo, Mixture model, Survival analysis, Weibull distribution

## Abstract

**Abstract:**

This study considered the problem of predicting survival, based on three alternative models: a single Weibull, a mixture of Weibulls and a cure model. Instead of the common procedure of choosing a single “best” model, where “best” is defined in terms of goodness of fit to the data, a Bayesian model averaging (BMA) approach was adopted to account for model uncertainty. This was illustrated using a case study in which the aim was the description of lymphoma cancer survival with covariates given by phenotypes and gene expression. The results of this study indicate that if the sample size is sufficiently large, one of the three models emerge as having highest probability given the data, as indicated by the goodness of fit measure; the Bayesian information criterion (BIC). However, when the sample size was reduced, no single model was revealed as “best”, suggesting that a BMA approach would be appropriate. Although a BMA approach can compromise on goodness of fit to the data (when compared to the true model), it can provide robust predictions and facilitate more detailed investigation of the relationships between gene expression and patient survival.

## Introduction

Modelling survival data plays an important role in the application of statistics in medicine and health science. In addition to a nonparametric formulation, there are many parametric models available for describing survival, including models based on a single distribution such as the Exponential and Weibull, mixture models based for example on mixtures of distributions and a mixture of susceptible and insusceptible individuals or so-called cure models which account for a fraction of the patients being cured from the disease. Given the wealth of models, the dilemma that is faced by many practitioners is the choice of a survival model.

The problem of model selection is abundant throughout the literature. This includes both covariate selection and choice of the model itself. Some of the methods are based on a series of significance tests while others fit more comprehensive models; some include prior information; some use analytic or approximate methods of estimation while others use Markov Chain Monte Carlo (MCMC) methods; different approaches use different optimisation or model comparison criteria such as Bayes factors (Raftery 1996). For example, McGrory and Titterington (2007) showed how variational techniques can be used to extend the deviance information criterion (DIC) to include the comparison of mixture models, while Basu and Tiwari (2010) used Bayes factors to compare the various model structures in breast cancer survival data.

Recently, Bonato et al. (2011) proposed Bayesian ensemble methods to obtain better survival prediction in high-dimensional gene expression data. Regardless of the method, the most common approach is to choose a single model based on the adapted optimisation or model choice criterion. However, if a single model is selected, then inferences are conditional on the selected model, and model uncertainty is ignored which often leads to excessively narrow or misleading inferences (Hjort and Claeskens 2003; Raftery et al. 1997). This difficulty can be overcome by combining the information provided by all suitable models into the analysis. The most common way of achieving this is to use a form of model averaging. From a Bayesian point of view, this averaging is applied such that the posterior distribution of the quantity of interest is obtained over the set of suitable models, weighted by the respective posterior model probabilities (Raftery 1996).

Draper (1995) and Raftery (1995) reviewed Bayesian model averaging (BMA) and the cost of ignoring model uncertainty. Madigan and Raftery (1994) also considered BMA by using Occam’s razor and Occam’s window approaches to reduce the number of candidate models. Yuan and Yin (2011) used model averaging procedures to make more robust inferences regarding the dose-finding design for phase I clinical trials. Pramana et al. (2012) focused on the case in which several parametric models are fitted to gene expression data and discussed model averaging techniques for the estimation of dose-response models.

In this paper, we consider the problem of predicting survival, based on three alternatives models; a single Weibull, a mixture of Weibulls and a cure model. The Weibull distribution is a popular parametric distribution for describing survival times (Dodson 1994). Given the variety of shapes that can be described by the probability density function (pdf) and the simple representation of the survival function, the Weibull distribution has been used very effectively for analysing lifetime data, particularly when the data are censored, which is very common in most life testing experiments (Collet 1994; Kundu 2008).

Given the nature of microarray data to describe biological systems and outcomes of patients, and the potential of these covariates to produce more precise inferences about survival, the use of a single parametric distribution to describe survival time may not be adequate. Microarray data may enable the description of several homogeneous subgroups of patients with respect to survival time. This paper therefore also considered a mixture of Weibull models for precise estimation and prediction of survival. Mixture models can be used to describe a population consisting of several disjoint groups, where each group is assigned its own distribution, weighted by the probability of an individual from the overall population belonging to that group. This model thus provides a convenient and flexible mechanism for identification and estimation of distributions which are not well modelled by any standard parametric family (Stephens 1997). In the study considered here, the mixture is assumed to comprise a known number of Weibull distributions, with potentially different parameters. Most approaches to the analysis of time to event data implicitly assume all individuals will experience the event of interest. However, there are situations when a proportion of individuals are not expected to experience the event of interest; that is, those individuals are often referred to as immune, cured or nonsusceptible (Ibrahim et al. 2001). To address this issue, cure rate models are considered, which are survival models incorporating a cure fraction. These models, which can be considered as a form of mixture model with one component degenerating to a point mass, extend the understanding of time to event data by allowing the formulation of more accurate and informative conclusions about the two groups of subjects.

Finally, instead of adopting the usual practice of choosing a single “best” model, where “best” is defined in terms of the probability of the model given the data, a BMA approach was adopted to account for model uncertainty in the prediction of the response. We illustrate the approach using a microarray dataset.

The paper is organised as follows. In Section “Methods”, we define BMA. The three competing models are described in a Bayesian framework in Section “Models”. The computational approach for estimation is also presented in this section. In the Section “Application to gene expression data”, we illustrate the model using a case study. The results are discussed further in Section “Discussion”.

## Methods

The key elements of BMA were discussed by Raftery (1995). He suggested weighting each model by the posterior model probabilities derived from a Bayesian analysis. Assume that there are *S* models being considered, for *s* = 1,2,…,*S*, each with parameter set *θ*_*s*_ based on data *D*. Let *Δ* be the quantity of interest; this could represent, for example, the posterior predictive distribution of *y*. Hence, the posterior distribution of *Δ* given data *D* (Hoeting et al. 1999) is 

 where *p*(*S* = *s* ∣ *D*) is the posterior probability of a particular model being true, defined as 

 where .

Here, *p*(*D* ∣ *S* = *s*) is the marginal likelihood of the data *D* given model *S* = *s* and *p*(*θ*_*s*_ ∣ *S* = *s*) is the prior density of *θ*_*s*_ given model *S* = *s*. *p*(*S* = *s*) is the prior probability that model *s* is the true model (Hoeting et al. 1999).

Given a model selection problem in which we have to choose between two models, the plausibility of the two different models *S*_1_ and *S*_2_ is assessed by the Bayes factor as the ratio of posterior model probabilities.

The main detractor from using Bayes factors is that they are, in general, difficult to compute. Raftery (1995) proposed using the Bayesian information criterion (BIC) (Schwarz 1978) as an approximation. Buckland et al. (1997) and Claeskens and Hjort (2008) discussed the utilization of BIC in BMA. Buckland et al. (1997) proposed simpler methods in which weights are based upon the penalised likelihood functions formed from the AIC (Akaike 1973).

The starting point for Burnham and Anderson’s model selection theory is the Kullback-Leibler (KL) information given by Burnham and Anderson (2002) and Claeskens and Hjort (2008): 

 where *f* represents the density function of the true and unknown model, *q* represents the density function of the model that is used to approximate *f*, and *θ*_*s*_ is a vector of the unknown parameters to be estimated. The notation *I*(*f* ∣ *q*) denotes the information lost when *q* is used to approximate *f* or the distance from *q* to *f*. For a given set of models, one can compare the KL information for each model and select the model that minimises the information loss across the considered set of models (Burnham and Anderson 2002;2004). However, in practice *I*(*f* ∣ *q*) cannot be computed since the true model *f* is unknown. Schwarz (1978) and Burnham and Anderson (2002) made the link between the KL information and likelihood theory, and showed that the expected KL information can be expressed as 

 where *p*(*D* ∣ *θ*_*s*_) is the likelihood, *d*_*s*_ is the number of parameters in the model and *n* is the number of uncensored observations in a survival context (Volinsky and Raftery 2000). A Laplace approximation, typically the BIC (Schwarz 1978), can be used to approximate *p*(*D* ∣ *S* = *s*) (Clyde 2000; Hoeting et al. 1999; Jackson et al. 2009; Yuan and Yin 2011): 1

Here  is the maximised log-likelihood of model *s*, which estimates goodness of fit of the data.

Schwarz (1978) and Burnham and Anderson (2002) proposed the likelihood of the model given the data, using  defined by 2

The BMA weight for the *s*^*th*^ model (Jackson et al. 2009; Yuan and Yin 2011) is therefore given by 

The BMA weight can be interpreted as the weight of the evidence that model *s* is true model given a set of *S* models. For the case in which there is no information about prior probabilities, we can let *p*(*S* = *s*) be equal for all candidate models (1/*S*), indicating no prior preference for any of the models (Jackson et al. 2009; Pramana et al. 2012). The model with the largest BMA weight will be considered as the best model. Therefore, *p*(*S* = *s* ∣ *D*) is also an approximation to the posterior probability of the model *s* being correct (Schwarz 1978). A smaller BIC value indicates a better model fit, accounting for model complexity.

Let  be the *j*^*th*^ simulated observation from the *s*^*th*^ model. Then the mean of *f* from the BMA model, (), can be calculated as follows 

 where *N* is the number of simulated observations and *w*_*s*_ = *p*(*S* = *s* ∣ *D*) is the BMA weight, defined previously.

## Models

### Weibull model

In this section, we define the Weibull model for analysing survival of patients in the context of human health. We confine ourselves to survival times that are the difference between a nominated start time and a declared failure (uncensored data) or a nominated end time (censored time). Let *T* be a nonnegative random variable for a person’s survival time and *t* be a realisation of the random variable *T*. Kleinbaum and Klein (2005) give some reasons for the occurrence of right censoring in survival studies, including termination of the study, drop outs, or loss to follow-up. For the censored observations, one could impute the missing survival times or assume that they are event-free. The former is often difficult, especially if the censoring proportion is large, and extreme imputation assumptions (such as all censored cases fail right after the time of censoring) may distort inferences (Leung et al. 1997; Stajduhar et al. 2009). In this study, we treat all censored cases as event-free regardless of observation time.

Initially, we assume that we observe survival times *t* of patients possibly from a heterogeneous population. The two-parameter Weibull density function for survival time is given by 

 for *α* > 0 and *γ* > 0, where *α* is a shape parameter and *γ* is a scale parameter (Ibrahim et al. 2001).

Since the logarithm of the Weibull hazard is a linear function of the logarithm of time, it is more convenient to write the model in terms of the parameterisation *λ* = log(*γ*) (Ibrahim et al. 2001), so that: 

 where *t* > 0, *α* > 0 and *γ*>0.

The corresponding survival function and the hazard function, using the *λ* parameterisation, are as follows: 

We now assume that we observe possibly right-censored data for *n* subjects; ***y*** = (*y*_1_,…,*y*_*n*_) where *y*_*i*_ = (*t*_*i*_,*δ*_*i*_) and *δ*_*i*_ is an indicator function such that (Marin et al. 2005a): 3

Let *x*_*ij*_ be the *j*^*th*^ covariate associated with *t*_*i*_ for *j* = 1,2,…,*p* + 1. In our case study, *x*_*ij*_ indicates the *p* gene expressions from DNA microarray data, and *x*_*i*0_ indicates the multi-category phenotype covariate. The data structure is as follows: 

The gene expression data can be included in the model through *λ* (Thamrin et al. 2013). Given that *λ* must be positive, one option is to include the covariates as follows: 4

Thus, the log-likelihood function becomes: 

We assume that (*α*,*λ*) are independent a priori (Marin et al. 2005a), and assign Gamma distributions. Thus, the priors are now given by: 

and we allow ***Σ*** to be diagonal with elements .

Diffuse priors are represented by large positive values for *σ*^2^, and small positive values for *u*_*α*_ and *v*_*α*_.

The joint posterior distribution of (*α*,***β***) is given by: 

MCMC analysis is performed by sampling from the conditional distributions of the parameters. The conditional distribution of *α* does not have an explicit form but can be sampled from MCMC algorithms such as Metropolis Hastings or slice sampling (Gilks et al. 1996).

### Weibull mixture model

We define the Weibull mixture model for analysing survival data. A mixture of *K* Weibull densities (Marin et al. 2005a) is defined by 5

where ***α*** = (*α*_1_,…,*α*_*K*_), ***γ*** = (*γ*_1_,…,*γ*_*K*_) are the parameters of each Weibull distribution and ***w*** = (*w*_1_,…,*w*_*K*_) is a vector of nonnegative weights which sum to one.

The corresponding survival function *S*(*t* ∣ *K*,***w***,***α***,***γ***) and hazard function *h*(*t* ∣ *K*,***w***,***α***,***γ***) are as follows: 

We now assume that we observe possibly right-censored data for *n* patients; ***y*** = (*y*_1_,…,*y*_*n*_) where *y*_*i*_ = (*t*_*i*_,*δ*_*i*_) and *δ*_*i*_ is an indicator function as described in Section “Weibull model”.

Let *x*_*ij*_ be the *j*^*th*^ covariate associated with patient *i*, for *j* = 1,2,…,*p*. In our application, *x*_*ij*_ could indicate, for example, the gene expressions. The covariates can be included in the model as follows (Farmomeni and Nardi 2010) 6

where ***x***_*i*_ = (*x*_*i*1_,…,*x*_*ip*_), *γ*_*m*_ = (*γ*_1*m*_,…,*γ*_*pm*_) and ***β***_*m*_ = (*β*_1*m*_,…,*β*_*pm*_), for *i* = 1,2,…,*n* and *m* = 1,2,…,*K*.

Thus, the likelihood function becomes: 

Here, the incomplete information is modelled via the survivor function, which reflects the probability that the patient was alive for duration greater than *t*_*i*_.

The following prior distributions are placed on the parameters ***w*** and ***α***: 

For a model without covariates, we employ the following prior for *γ*_*m*_. 

We chose small positive values for *u*_*α*_,*v*_*α*_,*u*_*γ*_,*v*_*γ*_ to express vague prior knowledge about these parameters and we set *ϕ* = 1 (Marin et al. 2005a). For a model with covariates, we employ a multivariate normal prior on ***β***_*m*_, so that 

 and we allow ***Σ*** to be diagonal with elements . Again, we express a vaguely informative prior by setting a large positive value for . The diagonal matrices were used here but this changed recently (Bhadra and Mallick 2013), so one may argue that a non-diagonal variace-covariance matrix may be more appropriate.

The model described in this section can be fitted using MCMC sampling with latent values *Z*_*i*_ to indicate component membership of the *i*^*th*^ observation (Diebolt and Robert 1994; Robert and Casella 2000). Since *w*_*m*_ = *Pr*(*Z*_*i*_ = *m*), we can write *Z*_*i*_ ∼ *M*(*w*_1_,…,*w*_*K*_). In this scheme, the *Z*_*i*_ are sampled by computing posterior probabilities of membership, and the other parameters are sampled from their full conditional distributions. This was implemented in the WinBUGS software package (Spiegelhalter et al. 2002).

The WinBUGS software (Lunn et al. 2000; Ntzoufras 2009; Spiegelhalter et al. 2002) is an interactive Windows version of the BUGS program for Bayesian analysis of complex statistical models using MCMC techniques.

Label switching, caused by non-identifiability of the mixture components, was dealt with post-MCMC using the reordering algorithm of Marin et al. (2005b). The algorithm proceeded by selecting the permutation of components at each iteration that minimised the vector dot product with the so-called “pivot”, a high density point from the posterior distribution. The MCMC output was then reordered according to each selected permutation. In this paper, the approximate maximum a posteriori (MAP) (i.e. the realization of parameters corresponding to the MCMC iterate that maximised the unnormalised posterior) was chosen as the pivot.

### Cure model

As in Section “Weibull model”, we observe time to the event of interest for *n* independent subjects, and we let (*t*_*i*_,*δ*_*i*_) denote the observed time and the event indicator for the *i*-th observation. Let *S*_1_(*t*) be the survivor function for the entire population, *S*^∗^(*t*) be the survivor function for the non-cured group in the population, and *π* be the cure rate function. Then the standard cure rate model is given by: 7

The commonly used parametric distributions include Exponential and Weibull for *S*^∗^(*t*).

As in Yakovlev and Tsodikov (1996), Chen et al. (1999) and Ibrahim et al. (2001), for an individual in a population, let *N* denote the number of latent variables. Assume that *N* has a Poisson distribution with mean *θ*. Let *Z*_*i*_, *i* = 1,…,*N* denote the random time, where *Z*_*i*_ are independently and identically distributed (i.i.d.) with a common distribution function *F*(*t*) = 1-*S*(*t*). Also, assume that *Z*_*i*_ are independent of *N*. The time to event can be defined by the random variable *Y* = min(*Z*_*i*_,0 ≤ *i* ≤ *N*), where *P*(*Z*_0_ = *∞*) = 1. Hence, the survival function for the population is given by 8

A corresponding cure fraction in model (8) is . We also know from (8) that the cure fraction is given by *S*_*pop*_(*∞*) = *P*(*N* = 0) = exp(-*θ*). As *θ* → *∞*, the cure fraction tends to 0, whereas as *θ*→0, the cure fraction tends to 1. Corresponding population density and hazard functions are  and *h*_*pop*_(*t*) = *θf*(*t*), respectively.

The proportional hazards structure with the covariates is modelled through *θ* (Chen et al. 1999; Ibrahim et al. 2001). The population survival function (8) can be written as 

where , and .

Following Chen et al. (1999) and Ibrahim et al. (2001), we construct the likelihood function. Suppose we have *n* subjects and we assume that the *N*_*i*_ are i.i.d with Poisson distributions with means *θ*_*i*_, *i* = 1,…,*n*. Let *Z*_*i*1_,…,*Z*_*iN*_ denote the times for the *N*_*i*_ competing causes, which are unobserved, and which have a cumulative distribution function, *F*(.). In this section, we will specify a parametric form for *F*(.) that is a Weibull distribution. Let ***ψ*** = (*α*,*λ*)^′^, where *α* is the shape parameter and *λ* is the scale parameter. We incorporate covariates for the cure rate model through the cure parameter *θ* and we have a different cure rate parameter, *θ*_*i*_, for each subject.

Let  denote the *k* x 1 vector of covariates for the *i*th subject, and let ***β*** = (*β*_1_,…,*β*_*k*_) denote the corresponding vector of regression coefficients. We relate *θ* to the covariates by . Let *t*_*i*_ denote the survival time for subject *i*, which is right censored, let *C*_*i*_ be the censoring time, and let *δ*_*i*_ be the censoring indicator, assuming 1 if *T*_*i*_ is a failure time and 0 if it is right censored. The observed data are *D* = (*n*,***t***,***δ***,***X***), where ***t*** = (*t*_1_,…,*t*_*n*_)^′^, ***δ*** = (*δ*_1_,…,*δ*_*n*_)^′^ and ***X*** = (*x*_1_,…,*x*_*n*_)^′^. The complete data are given by *D*_*c*_ = (*n*,***t***,***δ***,***X***,***N***), where ***N*** = (*N*_1_,…,*N*_*n*_)^′^. The complete-data likelihood function of the parameter (***ψ***,***β***) can be written as 9

Again, we assume independent priors for *β* and *ψ*, where *α*∼*Gamma*(*a*_*α*_,*b*_*α*_), *λ* ∼ *N*(***μ***_*λ*_,***Σ***_*λ*_) and ***β*** ∼ *N*(***μ***_*β*_,***Σ***_*β*_). We also assume *p*(*α*,*λ*) = *p*(*α* ∣ *δ*_0_,*τ*_0_)*p*(*λ*), , and the hyperparameters (*δ*_0_,*τ*_0_) are specified (Chen et al. 1999; Ibrahim et al. 2001).

Combining these specifications with the likelihood function (9), the joint posterior distribution of (*α*,*λ*,***β***) becomes 10

The joint posterior density of (*α*,*λ*,***β***) in equation (10) is analytically intractable because the integration of the joint posterior density is not easy to perform. Hence, inferences are based on MCMC simulation methods. We can use, for example, the Metropolis-Hastings algorithms or slice sampling to simulate samples of *α*,*λ* and ***β***. MCMC computations were implemented using the WinBUGS system (Spiegelhalter et al. 2002).

## Application to gene expression data

### DLBCL dataset

We applied the proposed method of model averaging across the three candidate survival models to a dataset containing gene expression of Diffuse Large B-cell Lymphoma (DLBCL). The dataset comprises gene expression measurements and survival times of patients with DLBCL (Rosenwald et al. 2002). DLBCL (Lenz et al. 2008) is a type of cancer of the lymphatic system in adults which can be cured by anthracycline-based chemotherapy in only 35 to 40 percent of patients (Rosenwald et al. 2002). In general, types of this disease are very diverse and their biological properties are largely unknown, meaning that this is a relatively difficult cancer to cure and prevent. Rosenwald et al. (2002) proposed that there are three phenotypes subgroups of patients of DLBCL: activated B-like DLBCL, germinal centre (GC)-B like and type III DLBCL. The GC B-like DLBCL is less dangerous than the others in the progression of the tumour; the activated B-like DLBCL is more active than the others and the type III DLBCL is the most dangerous in the progression of tumour (Alizadeh et al. 2000). These groups were defined using microarray experiments and hierarchical clustering. The authors showed that these phenotypes subgroups were differentiated from each other by distinct gene expressions of hundreds of different genes and had different survival time patterns. This dataset contains 219 patients with DLBCL, including 138 patient deaths during follow-up. Patients with missing values for a particular microarray element were excluded from all analyses involving that element.

Based on patterns of gene expression in biopsy specimens of the lymphoma, Rosenwald et al. (2002) analysed this dataset to predict the likelihood of patients’ survival after chemotherapy for DLBCL. By using a Cox proportional-hazards model, Rosenwald et al. (2002) identified five individual gene expressions which correlated with the survival after chemotherapy. These gene expressions are germinal center B-cell (GC-B), lymphoma node, proliferation, BMP6 and MHC. In this study, these five gene expressions are used as covariates for estimating survival times based on the three competing models in Section “Models”.

### Results

**Figure 1:**
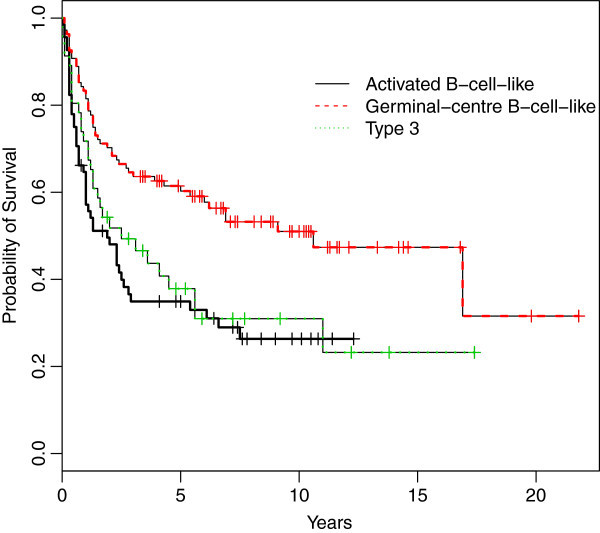
**Kaplan-Meier estimates of overall survival according to the gene-expression subgroups.**

As discussed in Section “Methods”, to account for model uncertainty, the model averaging technique which combines estimates from different survival models was carried out. This was accomplished through a weighted average of the survival considered in the analysis. First, we calculated the Kaplan-Meier estimates of overall survival according to the gene expression and the relation between the gene expression score and the subgroups phenotype of DLBCL. We confirmed that these phenotypes had different survival time patterns (Figure 1). Following this, we fitted the three models to all gene expression data and to the three phenotype subgroups. We then applied the BMA approach described in Section “Methods”. For each model, we ran the corresponding MCMC algorithm for 100 000 iterations, discarding the first 10 000 iterations as burn-in.

**Figure 2:**
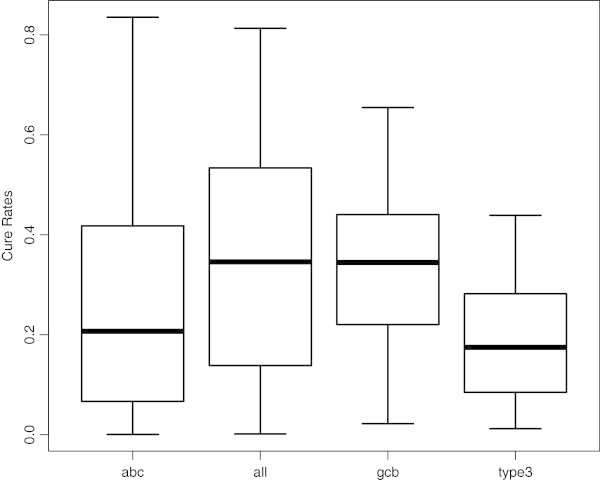
**Box-plots of the cure rates (posterior distribution of** ***π*** **) for the full DLBCL dataset, and to each of the three phenotypes (ABC, GCB and Type III).**

Table 1 shows the estimated posterior mean of the parameters, the 95% credible intervals (CI), the BIC values and the BMA weights for each of the fitted models for the whole dataset. The BMA weights reflect the relative posterior probability of the models. As can be seen from Table 1, for the Weibull model, there are three genes that substantially describe patients’ survival times, namely GC-B (*β*_1_), lymphoma node (*β*_2_) and MHC (*β*_5_). These three genes have a negative effect on the expected survival time. For the mixture model, GC-B (*β*_1_), lymphoma node (*β*_2_) and proliferation (*β*_3_) accounted for patients’ survival times in the first component. In the second component, GC-B (*β*_1_), lymphoma node (*β*_2_) and MHC signature (*β*_5_) substantially explained patients’ survival times. All these genes have negative effects on the expected survival time for their respective component. For the cure model, four of these genes substantially describe patients’ survival times, namely GC-B (*β*_1_), lymphoma node (*β*_2_), BMP6 (*β*_4_) and MHC (*β*_5_) signature. Three of these, namely GC-B (*β*_1_), lymphoma node (*β*_2_) and MHC signature (*β*_5_), have a negative effect on the expected survival time. Under the cure model, approximately 33.8% of the patients are cured of DLBCL (Figure 2).

**Table 1:** **The estimated posterior mean of the parameters, the 95% credible intervals (CI), the BIC values and the BMA weights for each of the fitted models for the full DLBCL dataset**

Model	Parameter	Mean	95% CI	BIC	Weight
Weibull	*α*	0.7305	(0.626,0.840)	687.0953	0.0009
	*β*_0_	-1.578	(-1.84, -1.33)		
	*β*_1_	-0.3446	(-0.516, -0.172)		
	*β*_2_	-0.2844	(-0.454, -0.116)		
	*β*_3_	0.2097	(-0.049, 0.468)		
	*β*_4_	0.3292	(0.115, 0.537)		
	*β*_5_	-0.3019	(-0.488, -0.112)		
Mixture	*α*_1_	4.029	(2.411, 6.631)	734.0054	≈0
	*α*_2_	0.7707	(0.662, 0.885)		
	*β*_01_	6.857	(5.479, 8.205)		
	*β*_02_	-1.724	(-2.007, -1.457)		
	*β*_11_	-11.62	(-12.88, -10.35)		
	*β*_12_	-0.3956	(-0.575, -0.216)		
	*β*_21_	-2.087	(-3.54, -0.689)		
	*β*_22_	-0.3172	(-0.495, -0.143)		
	*β*_31_	-2.241	(-3.425, -1.059)		
	*β*_32_	0.1972	(-0.064, 0.461)		
	*β*_41_	-0.2849	(-1.434, 0.854)		
	*β*_42_	0.3594	(0.141, 0.574)		
	*β*_51_	-0.7928	(-2.107, 0.477)		
	*β*_52_	-0.3102	(-0.500, -0.115)		
	*π*_1_	0.01992	(0.002, 0.053)		
	*π*_2_	0.9801	(0.946, 0.997)		
Cure	*α*	0.9884	(0.828, 1.145)	673.1359	0.9991
	*β*_0_	0.1611	(-0.124, 0.560)		
	*β*_1_	-0.3151	(-0.484, -0.144)		
	*β*_2_	-0.2821	(-0.451, -0.115)		
	*β*_3_	0.189	(-0.070, 0.442)		
	*β*_4_	0.3303	(0.118, 0.539)		
	*β*_5_	-0.3039	(-0.490, -0.112)

This is clearly exhibited in Table 1, which shows that the cure model has the largest posterior model probability (or BMA weight). To evaluate the model fit, a comparison of predicted values under the models and of the observed data was carried out.

Table 2 shows the 95% credible intervals (CI), BIC values and the BMA weights for each of the models based on phenotype for the DLBCL dataset. In general, for all phenotypes, the mixture model is not favourable as its weight is approximately equal to zero and it has the largest BIC value. On the other hand, the BIC values of the other two models are close to each other, suggesting a combination of these two models in order to account for the uncertainty in the prediction of survival.

**Table 2:** **The estimated posterior mean of parameters, the 95% CI, BIC values and the BMA weights for each of the models based on phenotype for the DLBCL dataset**

Phenotype	Model	Variable	Parameter	Mean	95% CI	BIC	Weight
GCB	Weibull		*α*	0.692	(0.5365, 0.8595)	341.212	0.497
		Intercept	*β*_0_	-1.649	(-2.185, -1.17)		
		GCB	*β*_1_	-0.179	(-0.5859, 0.239)		
		Lymphoma	*β*_2_	-0.118	(-0.3958, 0.1607)		
		Proliferation	*β*_3_	0.459	(-0.0306, 0.934)		
		BMP6	*β*_4_	0.414	(0.01773, 0.809)		
		MHC	*β*_5_	-0.325	(-0.6389, -0.01228)		
	Mixture		*α*_1_	4.252	(2.591, 7.175)	377.759	≈0
			*α*_2_	0.816	(0.6209, 1.032)		
		Intercept	*β*_01_	6.491	(5.246, 7.781)		
			*β*_02_	-2.152	(-2.798, -1.567)		
		GCB	*β*_11_	-11.81	(-13.05, -10.53)		
			*β*_12_	-0.030	(-0.5104, 0.4592)		
		Lymphoma	*β*_21_	-1.839	(-3.082, -0.6744)		
			*β*_22_	-0.134	(-0.48, 0.2254)		
		Proliferation	*β*_31_	-2.165	(-3.313, -0.9932)		
			*β*_32_	0.588	(-0.07796, 1.407)		
		BMP6	*β*_41_	-0.242	(-1.357, 0.8482)		
			*β*_42_	0.654	(0.17, 1.161)		
		MHC	*β*_51_	-0.629	(-1.993, 0.5117)		
			*β*_52_	-0.382	(-0.7687, -0.002227)		
			*ϕ*_1_	0.090	(0.02007, 0.1863)		
			*ϕ*_2_	0.91	(0.8137, 0.9799)		
	Cure		*α*	0.845	(0.6075, 1.1)	341.188	0.503
		Intercept	*β*_0_	0.604	(-0.3556, 3.394)		
		GCB	*β*_1_	-0.173	(-0.5754, 0.2402)		
		Lymphoma	*β*_2_	-0.116	(-0.3891, 0.1579)		
		Proliferation	*β*_3_	0.433	(-0.0522, 0.9041)		
		BMP6	*β*_4_	0.396	(-0.0007, 0.788)		
		MHC	*β*_5_	-0.330	(-0.6422, -0.0209)		
ABC	Weibull		*α*	0.894	(0.695, 1.115)	215.564	0.013
		Intercept	*β*_0_	-1.86	(-2.562, -1.217)		
		GCB	*β*_1_	-0.509	(-0.9948, -0.03871)		
		Lymphoma	*β*_2_	-0.626	(-0.9568, -0.3099)		
		Proliferation	*β*_3_	-0.487	(-1.118, 0.1422)		
		BMP6	*β*_4_	0.645	(0.2725, 1.021)		
		MHC	*β*_5_	-0.479	(-0.7955, -0.1598)		
	Mixture		*α*_1_	2.427	(1.083, 4.152)	256.552 ‘	≈0
			*α*_2_	0.960	(0.7525, 1.189)		
		Intercept	*β*_01_	6.636	(5.301, 7.959)		
			*β*_02_	-2.572	(-3.346, -1.865)		
		GCB	*β*_11_	-12.11	(-13.36, -10.86)		
			*β*_12_	-0.925	(-1.438, -0.4356)		
		Lymphoma	*β*_21_	-3.155	(-4.578, -1.75)		
			*β*_22_	-0.768	(-1.114, -0.4341)		
		Proliferation	*β*_31_	-2.377	(-3.561, -1.188)		
			*β*_32_	-0.480	(-1.099, 0.1353)		
		BMP6	*β*_41_	0.079	(-1.064, 1.232)		
			*β*_42_	0.690	(0.3249, 1.061)		
		MHC	*β*_51_	-0.644	(-1.919, 0.6499)		
			*β*_52_	-0.515	(-0.8176, -0.2047)		
			*ϕ*_1_	0.037	(0.0046, 0.09883)		
			*ϕ*_2_	0.963	(0.9012, 0.9953)		
	Cure		*α*	1.189	(0.8906, 1.483)	206.961	0.987
		Intercept	*β*_0_	0.019	(-0.6417, 0.7362)		
		GCB	*β*_1_	-0.432	(-0.8874, 0.01376)		
		Lymphoma	*β*_2_	-0.587	(-0.905, -0.2867)		
		Proliferation	*β*_3_	-0.484	(-1.076, 0.1012)		
		BMP6	*β*_4_	0.607	(0.2557, 0.9631)		
		MHC	*β*_5_	-0.446	(-0.7481, -0.1346)		
Type III	Weibull		*α*	0.834	(0.5958, 1.101)	162.27	0.538
		Intercept	*β*_0_	-1.75	(-2.736, -0.9093)		
		GCB	*β*_1_	-0.404	(-1.028, -0.19)		
		Lymphoma	*β*_2_	-0.274	(-0.7404, 0.1644)		
		Proliferation	*β*_3_	0.506	(-0.0897, 1.084)		
		BMP6	*β*_4_	0.017	(-0.5301, 0.5206)		
		MHC	*β*_5_	-0.199	(-0.6839, 0.3098)		
	Mixture		*α*_1_	11.82	(8.609, 15.14)	196.271	≈0
			*α*_2_	0.596	(0.43, 0.7757)		
		Intercept	*β*_01_	6.002	(3.682, 8.336)		
			*β*_02_	-5.005	(-7.19, -2.812)		
		GCB	*β*_11_	-9.32	(-12.02, -6.611)		
			*β*_12_	0.564	(0.1829, 1.004)		
		Lymphoma	*β*_21_	-2.913	(-5.716, -0.02716)		
			*β*_22_	-0.558	(-1.015, -0.1525)		
		Proliferation	*β*_31_	-2.021	(-4.547, 0.455)		
			*β*_32_	0.893	(0.3455, 1.515)		
		BMP6	*β*_41_	0.320	(-2.466, 3.373)		
			*β*_42_	0.140	(-0.2735, 0.5384)		
		MHC	*β*_51_	-0.336	(-2.733, 2.323)		
			*β*_52_	-0.293	(-0.7741, 0.1504)		
			*ϕ*_1_	0.072	(0.0108, 0.1805)		
			*ϕ*_2_	0.928	(0.8195, 0.9891)		
	Cure		*α*	0.969	(0.6534, 1.339)	162.578	0.462
		Intercept	*β*_0_	0.989	(-0.5077, 4.153)		
		GCB	*β*_1_	-0.349	(-0.973, -0.25)		
		Lymphoma	*β*_2_	-0.269	(-0.7375, 0.1687)		
		Proliferation	*β*_3_	0.502	(-0.0955, 1.084)		
		BMP6	*β*_4_	0.046	(-0.4801, -0.1801)		
		MHC	*β*_5_	-0.183	(-0.6625, 0.3207)		

From Tables 1 and 2, we can see that the Weibull model is better than a two-component Weibull mixture model.

**Figure 3:**
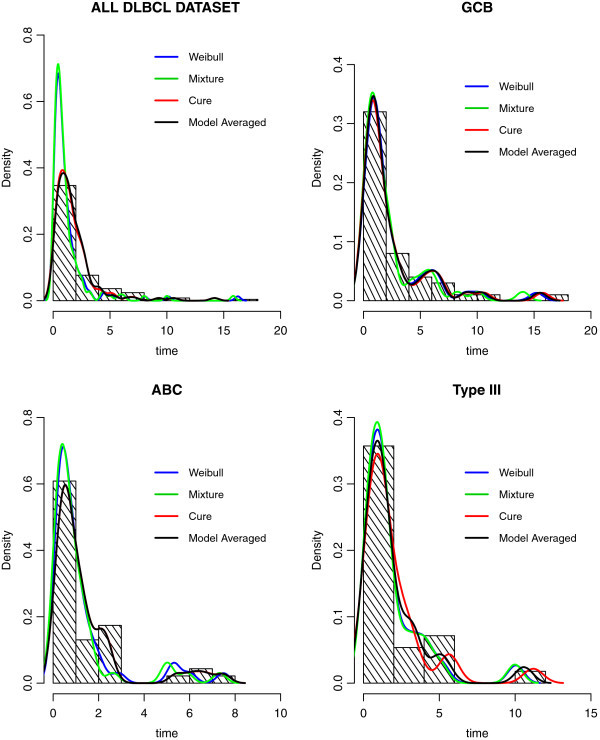
**The posterior densities of the three models and the model averaged density for the full DLBCL dataset and each of the three phenotypes.** For comparison, the observed data is also represented as a histogram.

As can be seen in Figure 3, in the full DLBCL dataset, the predicted curve for the cure model is quite close to the observed data, suggesting a good fit of the data. Specifically, in this model, 94.3% of observed survival times in the dataset fall in the corresponding 95% posterior prediction intervals. As expected, this is quite similar to the result obtained from model averaging (91.9%) (Table 3).

**Table 3:** **The percentage of observed values that lay in the corresponding 95% posterior prediction interval for the individual models and BMA model based on the full DLBCL dataset and each of the three phenotypes**

Model	All DLBCL	GCB	ABC	Type III
Weibull	87.5	90	89.1	89.3
Mixture	85.9	88	86.9	82.1
Cure	94.3	92	91.3	85.7
BMA	91.9	94	93.4	92.8

Furthermore, in the GCB phenotype, the genes corresponding to the BMP6 (*β*_4_) and MHC signature (*β*_5_) in the Weibull model and MHC signature (*β*_5_) in the cure model substantially affect patients’ survival time. In the ABC phenotype, in the Weibull model, with the exception of proliferation (*β*_3_), all genes were involved substantially in the description of patients’ survival and lymphoma node (*β*_2_), BMP6 (*β*_4_) and MHC signature (*β*_5_) are potentially important prognostic factors for predicting survival in the cure model. For the type III phenotype, the GC-B gene (*β*_1_) in both models and only the BMP6 gene (*β*_4_) in the cure model are substantial in explaining the survival times of the patients.

Under the cure model, in the GCB phenotype, approximately 33.2% of the patients are estimated to be cured of DLBCL. In the ABC and type III phenotypes, the respective cure rates are approximately 26.6% and 18.7% (Figure 2).

The results of the posterior densities prediction for the individual models and the model averaged prediction based on these three phenotypes are presented in Figure 3. In comparison to other models, the mixture model fitted the data poorly for each phenotype. In detail, using model averaging, for the GCB phenotype, 94% of the observed survival times in the dataset lie in the respective 95% prediction intervals. For the other two phenotypes, namely the ABC and the type III, 93.4% and 92.8% of the observed survival times in the dataset are in the corresponding 95% prediction intervals, respectively (Table 3).

## Discussion

This study has adopted a Bayesian model averaging approach to account for model uncertainty in the prediction of survival. The case study that we considered involved lymphoma cancer survival, with covariates given by phenotypes and gene expressions. Here, we proposed three competing models and used BMA to combine these models to account for model uncertainty.

Overall, the results of this study indicate that if using the full dataset without further grouping, selecting a single model that best fits the data was adequate. The reason is that there is clear support for one model (i.e. only one model has a relatively larger BIC value and dominates based on this criterion). However, the results were different when the model selection process took into account the phenotype subgroups of the patients. A single model appeared to be inadequate. This was due to the fact that the values of BIC for the Weibull and the cure had nearly equal weight, indicating the absence of a dominant model based on this criterion and the presence of uncertainty issues in the model selection. As suggested and shown in this study, BMA was used to address this problem. The applicability of BMA was also associated with the smaller sample size in each phenotype subgroup (Annest et al. 2009; Volinsky et al. 1997; Yeung et al. 2005).

This study also revealed that in each phenotype, the expression and number of predictor genes substantially describing the survival times of the patients varied across models. Overall, in both of the favourable models, none of the genes were identified consistently as substantial predictors for the patients’ survival. For example, in the Weibull model, the MHC and BMP genes in the GCB and ABC phenotypes and the GCB genes in the ABC and Type III phenotypes were important predictors of survival. In contrast, in the cure model, BMP was substantially associated with predicted survival in the ABC and Type III phenotypes. For both models, only three genes i.e. lymphoma node, BMP6 and MHC signature in the ABC phenotype were highly associated with the survival times of the patients.

This study has indicated that the application of BMA to combine competing models overcomes the problem of model uncertainty. Comparison of different survival models has allowed the identification and analysis of more detailed relationships between gene expressions in given phenotypes and the survival times of the patients. An advantage of BMA is more accurate and precise prediction of patient survival. However, this study only involved three candidate models. More models can be obviously included in the analysis. This study has also focused on the marginal likelihood *p*(*D* ∣ *Q*_*s*_) estimation methods based on the Laplace approximation. However, other approaches are also possible. Indeed marginal likelihood estimation is possible using nested sampling (Skilling 2006), where the marginal likelihood is viewed as the expectation, with respect to the prior, of the likelihood. Another generic approach is Chib’s method (Chib 1995), which can be applied to output from the Gibbs sampler. Applying BMA to other datasets or other applications is desired to obtain robust predictions.
